# The methyl donor S-adenosyl methionine reverses the DNA methylation signature of chronic neuropathic pain in mouse frontal cortex

**DOI:** 10.1097/PR9.0000000000000944

**Published:** 2021-07-13

**Authors:** Lucas Topham, Stephanie Gregoire, HyungMo Kang, Mali Salmon-Divon, Elad Lax, Magali Millecamps, Moshe Szyf, Laura Stone

**Affiliations:** a Alan Edwards Centre for Research on Pain, McGill University, Montreal, QC, Canada; b Faculty of Dentistry, McGill University, Montreal, QC, Canada; c Department of Molecular Biology, Ariel University, Ariel, Israel; d Department of Pharmacology and Therapeutics, McGill University, Montreal, QC, Canada; e Adelson School of Medicine, Ariel University, Ariel, Israel; f Sackler Program for Epigenetics and Psychobiology, McGill University, Montreal, QC, Canada; g Department of Anesthesiology, McGill University, Montreal, QC, Canada; h Department of Neurology and Neurosurgery, McGill University, Montreal, QC, Canada; i Department of Anesthesiology, Faculty of Medicine, University of Minnesota, Minneapolis, MN, USA

## Abstract

Chronic pain is associated with persistent but reversible structural and functional changes in the prefrontal cortex (PFC). This stable yet malleable plasticity implicates epigenetic mechanisms, including DNA methylation, as a potential mediator of chronic pain–induced cortical pathology. We previously demonstrated that chronic oral administration of the methyl donor S-adenosyl methionine (SAM) attenuates long-term peripheral neuropathic pain and alters global frontal cortical DNA methylation. However, the specific genes and pathways associated with the resolution of chronic pain by SAM remain unexplored.

**Objective:**

To determine the effect of long-term therapeutic exposure to SAM on the DNA methylation of individual genes and pathways in a mouse neuropathic pain model.

**Methods:**

Male CD-1 mice received spared nerve injury or sham surgery. Three months after injury, animals received SAM (20 mg/kg, oral, 3× a week) or vehicle for 16 weeks followed by epigenome-wide analysis of frontal cortex.

**Results:**

Peripheral neuropathic pain was associated with 4000 differentially methylated genomic regions that were enriched in intracellular signaling, cell motility and migration, cytoskeletal structure, and cell adhesion pathways. A third of these differentially methylated regions were reversed by SAM treatment (1415 regions representing 1013 genes). More than 100 genes with known pain-related function were differentially methylated after nerve injury; 29 of these were reversed by SAM treatment including *Scn10a, Trpa1, Ntrk1,* and *Gfap*.

**Conclusion:**

These results suggest a role for the epigenome in the maintenance of chronic pain and advance epigenetic modulators such as SAM as a novel approach to treat chronic pain.

## Abstract 2

Supplemental Digital Content is Available in the Text.

Chronic administration of S-adenosylmethionine reverses neuropathic pain–induced changes in DNA methylation in the mouse frontal cortex.

## 1. Introduction

The prefrontal cortex (PFC) is essential for executive functioning; it synthesizes the diverse range of input that the brain receives and is a crucial area for processing nociceptive and aversive stimuli.^42,47,77^ The PFC is part of cortical and subcortical networks that are activated by pain^6,29^ and that mediate its affective-motivational and cognitive components.^9,10,44^ These higher-order aspects of pain can exacerbate an individual's perception of pain and contribute to pain-related comorbidities and reduced quality of life.^8,13,46,51^ As a result of chronic pain, the PFC undergoes structural and anatomical changes over time resulting in the loss of gray matter, reorganization of synaptic connections, and changes in gene expression.^2,20,31,41,61^

Reduced PFC gray matter has been demonstrated in multiple pain conditions in humans and animal models.^7,24,28,35,55,67,76^ Interestingly, treatments that attenuate pain are capable of reversing chronic pain–induced losses of cortical thickness or gray matter in the PFC.^54,56,57,60^ Gene expression in the PFC is also heavily modified in response to chronic pain with genome-wide changes in mRNA expression detected in long-term neuropathic pain.^2,20,49^

Long-term regulation of gene expression can be embedded by epigenetic mechanisms, including histone modification and DNA methylation. Epigenetic regulation allows for stable control of gene expression to establish cell-type–specific phenotypes and dynamic alterations in gene expression in response to experiential and environmental signals, without modifying the underlying genetic sequence. DNA methylation is an enzymatically catalyzed covalent addition of methyl residues to cytosine nucleotides. DNA methylation in the promoter region of a gene or critical enhancer positions typically results in epigenetic repression of gene expression.^25,33^ DNA methylation is catalyzed by DNA methyltransferases (DNMTs) that transfer a methyl moiety from the methyl donor S-adenosyl methionine (SAM) to cytosine bases in DNA.^37,78^ S-adenosyl methionine is marketed as a nutritional supplement for a range of pain-related or comorbid conditions including depression, cognitive deficits, migraine, back pain, and osteoarthritis.^32,43,45^ DNA methylation may mediate, in part, the lasting changes in gene expression associated with chronic pain.

A number of studies have demonstrated that chronic pain is accompanied by changes in DNA methylation throughout pain pathways including dorsal root ganglion,^26,50^ spinal cord,^71^ and brain including PFC.^27,39,63,64^ Studies examining promoter region DNA methylation found gene-specific changes that correlated to mRNA transcript levels^39^ and were dynamically regulated up to 1 year postinjury.^64^ Moreover, successful attenuation of pain by environmental enrichment reduced global PFC DNA methylation levels in the PFC.^63^ However, the individual genes and biological systems that are associated with effective treatment of pain have not been explored. Identification of these systems will provide insight into the mechanisms by which chronic pain becomes embedded in the epigenome and how the epigenome can be targeted therapeutically.

We recently demonstrated that systemic chronic treatment with the methyl donor SAM attenuated the sensory and cognitive impact of peripheral nerve injury in mice.^27^ Here, we demonstrate that SAM treatment counteracts nerve injury–related changes in DNA methylation and is associated with functional pathways that are activated by and relevant to chronic pain.

## 2. Materials and methods

### 2.1. Animals

Male CD-1 mice (Charles River Laboratories, St-Constant, QC, Canada) were received at 6 to 8 weeks of age and housed 3 to 4 per cage on a 12-hour light/dark cycle in a temperature-controlled room in ventilated polycarbonate cages (Allentown, Allentown, NJ) with corncob bedding (7097, Teklad Corncob Bedding; Envigo, United Kingdom) and cotton nesting squares for enrichment. Mice were given access to food (2092X Global Soy Protein-Free Extruded Rodent Diet, Irradiated) and water ad libitum. Animals were habituated to the housing conditions for at least 1 week before any experimental interventions.

All experiments were approved by the Animal Care Committee at McGill University and conformed to the ethical guidelines of the Canadian Council on Animal Care and the guidelines of the Committee for Research and Ethical Issues of the International Association for the Study of Pain.^79^

### 2.2. Neuropathic pain model: spared nerve injury

Animals were randomly assigned to receive either spared nerve injury (SNI) or sham surgery. Nerve injury was induced using the SNI model of neuropathic pain, as adapted for mice^18,59^ at 10 to 12 weeks of age. Under deep isoflurane anesthesia, an incision was made on the medial surface of the thigh, exposing the 3 branches of the sciatic nerve. The tibial and common peroneal branches were tightly ligated with 6-0 silk (Ethicon) and sectioned distal to the ligation. Sham surgery consisted of exposing the nerve without damaging it.

### 2.3. Drug treatment: chronic administration of S-adenosylmethionine

Three months after SNI or sham surgery, animals were randomly assigned to receive saline vehicle or SAM (kind gift of Life Science Labs Supplements, LLC, Lakewood, NJ) treatment for 4 months. A solution of SAM (20 mg/kg) was freshly prepared each treatment day in 0.9% NaCl, and each animal received an oral administration of 8 μL 3 times per week for 4 months. The chosen dose and the protocol used were informed by previous studies.^14,21,48^

### 2.4. Behavioural assessment of mechanical sensitivity

Baseline behavioral assessments were performed 3 months after SNI or sham surgery to confirm the development of neuropathic pain. Behavioural testing group sizes were as follows: Sham-Vehicle: n = 6, Sham-SAM: n = 6, SNI-Vehicle: n = 7, and SNI-SAM: n = 8. Mechanical sensitivity was reassessed every 2 weeks across the 4 months of treatment. After a 1-hour habituation period to the testing apparatus, von Frey filaments (Stoelting Co, Wood Dale, IL) were applied to the plantar surface of the hind paw until filaments were bent for either 3 seconds or the animal withdrew the hind paw. Mechanical sensitivity was determined as the 50% withdrawal threshold using the up-down method.^16^ The stimulus intensity ranged from 0.04 to 4.0 g. The experimenter was blind to treatment group.

### 2.5. Isolation of prefrontal cortex, DNA capture, and bisulfite sequencing

Tissue processing, DNA capture, and bisulfite sequencing protocols were performed according to previously described protocols.^64^ Capture probes were designed to target enhancer and promoter regions of the mouse genome based on H3K4me1-associated and H3K4me3-associated regions. Genomic coordinates of bisulfite capture sequencing probes can be found in Supplemental Table 1 (available at http://links.lww.com/PR9/A115). In brief, after completion of SAM treatment, mice underwent isoflurane anesthesia and decapitation, and mouse frontal cortex was isolated as previously described.^64^ Genomic DNA was extracted, and bisulfite capture sequencing was performed according to the Roche SeqCap Epi Developer M Enrichment system. DNA sequencing was performed by Genome Quebec on the Illumina HiSeq 2500 following Illumina guidelines. DNA methylation analysis group sizes were as follows: Sham-Vehicle: n = 3, Sham-SAM: n = 3, SNI-Vehicle: n = 4, and SNI-SAM: n = 4.

### 2.6. DNA methylation preprocessing

DNA methylation preprocessing was performed as described previously.^64^ In brief, capture sequencing data were preprocessed using the McGill University Genome Quebec Innovation Centre GenPipes Methyl-Seq Pipeline. The pipeline proceeds through Trimmomatic, Bismark Align, Picard Deduplication, and Bismark Methylation Call.^11,34^ Sequence reads were aligned based on the mm10 genome. Collected sequence data were filtered to remove a blacklist of regions identified to have anomalous, unstructured, and high-signal/read counts in next-generation sequencing.^3^ The blacklist used was specific for mm10, and the BED file can be found in Supplemental Table 2 (available at http://links.lww.com/PR9/A116).

### 2.7. Differential methylation analysis

Differential methylation analysis was performed using R and the MethylKit package.^1^ For a CpG site to be analysed, it was required to be sequenced to a minimum depth of at least 10 reads in each sample. Coverage values between samples were normalized using a scaling factor derived from differences between the median of coverage distributions. Tiling windows were used to summarize methylation information over the genome. Tiles were set to be 250 base pairs (bps) in length and advance in 125 bp steps. Methylated and unmethylated cytosine counts within each tile were summed to provide an overall methylation proportion within the tile. Only CpGs annotated to a promoter region, defined as 2000 bp upstream or 200 bp downstream from the transcription start site, were selected for analysis. MethylKit uses logistic regression to model a log odds ratio based on methylation proportion within each tile. Calculated *P* values are adjusted using the SLIM method.^70^

Tiles were defined as being differentially methylated between groups if the adjusted *P* value was <0.1 and had a difference in methylation >5%. Although the selection of a threshold of 0.1 carries greater risk of false positives than the more typical threshold of 0.05, it is preferable for the exploratory purposes of this study. Differentially methylated tiles were further divided into hypermethylated or hypomethylated. In comparisons between groups, the control group acts as the reference point, ie, Control: Sham-Vehicle and Experimental: SNI-Vehicle. If the experimental group tile methylation is 5% or larger than the control, the tile is considered to be hypermethylated, and if the experimental tile methylation is reduced by −5% or more compared with control, the tile is considered to be hypomethylated.

Tiles were annotated to their associated genes and genomic regions using the annotatePeaks function of the ChIPseeker^75^ R package, using a TxDb object created from the Gencode “mmusculus_gene_ensembl” BioMart database. Each tile position was annotated and tracked individually throughout the analysis. For homology conversion, gene symbols from non-mouse species were converted to known mouse homologs based on the Mouse Genome Database (MGD)—Vertebrate Homology, Mouse Genome Informatics, The Jackson Laboratory, Bar Harbor, Maine.^12^

The data discussed in this publication have been deposited in NCBI's Gene Expression Omnibus^22^ and are accessible through GEO Series accession number GSE162016.

### 2.8. Validation by pyrosequencing

Targeted bisulfite sequencing was used to validate identified differentially methylation regions, performed by Zymo Research (Irvine, California) as per their MethylCheck pipeline. In brief, primers were designed to flank target regions, bisulfite conversion was performed, and barcoded samples underwent Illumina sequencing. Estimated methylation levels were provided for each CpG within the selected target regions. Group sizes were as follows: Sham-Vehicle: n = 6, Sham-SAM: n = 6, SNI-Vehicle: n = 6, and SNI-SAM: n = 9.

### 2.9. Statistical analysis

One-way analysis of variance with the Holm–Sidak post hoc test was used to detect group differences in the von Frey mechanical sensitivity behavioural assay. Pairwise comparisons of tiles between experimental groups used logistic regression to model the log odds ratio of methylation proportion per tile. Analyses were corrected for multiple comparisons using the SLIM method.^70^ The hypergeometric test was used to test for significant enrichment of pain-related genes.

### 2.10. Gene ontology analysis

Differentially methylated genes were submitted to g:Profiler,^52^ a web-based gene ontology tool. The Gene Ontology (GO) Biological Process and Molecular Function databases were selected as ontology databases. Ontology *P* values were adjusted using the g:SCS correction for multiple comparisons,^53^ and an ontology was considered enriched at an adjusted *P* value < 0.05.

## 3. Results

**Figure 1.**
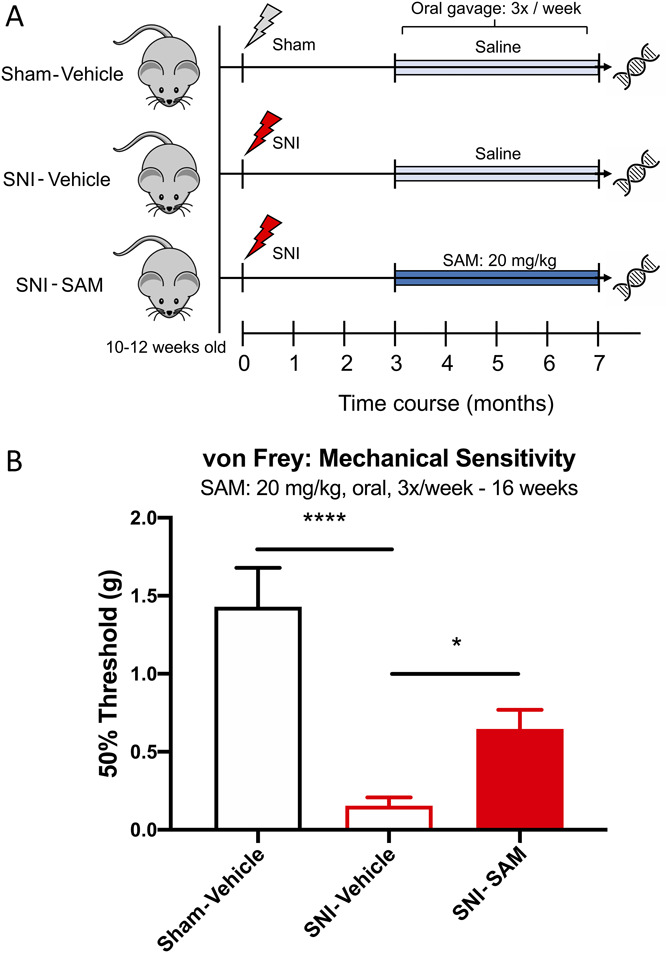
Chronic systemic administration of S-adenosyl methionine attenuates peripheral neuropathic pain. (A) Experimental paradigm of SAM treatment. Animals underwent either sham or SNI surgery and at 3 months postinjury received either saline or SAM (20 mg/kg) through oral gavage 3x per week for 4 months. Frontal cortex was extracted 7 months postinjury for DNA bisulfite sequencing. (B) Repeated administration of SAM over a 4-month period attenuates SNI-related mechanical hypersensitivity compared with vehicle-treated animals. Sham-Vehicle: n = 6, SNI-Vehicle: n = 7, and SNI-SAM: n = 8. One-way ANOVA with the Holm–Sidak post hoc test. *P < 0.05, ****P < 0.0001. Adapted from Gregoire et al. 2016^27^. ANOVA, analysis of variance; SNI, spared nerve injury; SAM, S-adenosyl methionine.

As previously reported, chronic administration of the methyl donor SAM beginning 3 months post-SNI injury decreases mechanical hypersensitivity in the ipsilateral injured paw compared with saline vehicle-treated animals^27^ (Fig. 1). S-adenosyl methionine had no effect on mechanical sensitivity in Sham-operated control animals^27^ (Supplemental Fig. 1A, available at http://links.lww.com/PR9/A124).

### 3.1. SAM treatment is associated with a reversal of spared nerve injury–induced differential methylation

**Figure 2.**
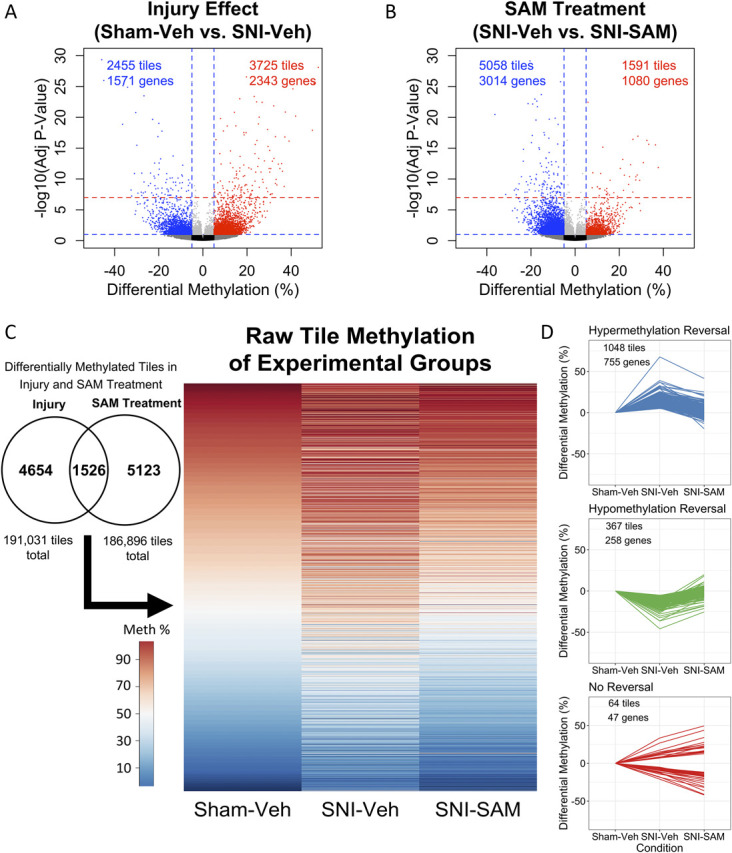
Reversal of chronic pain–induced DNA methylation after SAM treatment. Volcano plots depict the magnitude and statistical significance of promoter region tile differential methylation during (A) injury and (B) SAM treatment of injury. Tiles are displayed for positive or negative methylation with the control group as the reference point (x-axis), against –log10(adjusted *P* value; *y*-axis). Blue horizontal dashed line indicates an adjusted *P* value threshold of 0.1, and red horizontal dashed line indicates an adjusted *P* value threshold of 1 × 10^−7^. The blue vertical dashed line indicates methylation difference thresholds of 5% and −5%. Red: hypermethylated; blue: hypomethylated; dark gray: nonsignificant but with methylation differences of >5%; light gray: significant but with methylation differences of <5%; and black = not different. (C) A total of 1526 tiles are differentially methylated in both Injury and SAM treatment. Of these 1526 tiles, 1415 tiles that were differentially methylated by injury (Sham-Veh vs SNI-Veh) were reversed towards uninjured levels by SAM treatment (SNI-Veh to SNI-SAM). (D) Individual tiles showed a reversal of hypermethylation, a reversal of hypomethylation or no reversal after SAM treatment. Sham-Vehicle: n = 3, SNI-Vehicle: n = 4, and SNI-SAM: n = 4. SNI, spared nerve injury; SAM, S-adenosyl methionine; Veh: vehicle.

Changes in promoter region methylation in the mouse frontal cortex in response to SNI at multiple time points postinjury were previously reported.^64^ Here, we examined genome-wide promoter DNA methylation postinjury and post-SAM administration to determine the effect of SAM on injury-related changes in the frontal cortex. To determine the injury effect, SNI-Vehicle animals were compared against Sham-Vehicle animals 7 months postinjury, with 191,013 tiling regions covering 22,683 unique gene promoters. We detected 3725 hypermethylated tiles and 2455 hypomethylated tiles, representing 2343 and 1571 unique genes, respectively, whose state of methylation was altered by SNI (Fig. 2A). Of these, 157 hypermethylated tiles and 95 hypomethylated tiles displayed robust differential methylation (adjusted *P* value < 1 × 10^−7^).

To determine the SAM treatment effect, SNI-SAM animals were compared with SNI-Vehicle animals, with 186,896 tiling regions evaluating 22,530 unique gene promoters. We detected 1591 hypermethylated tile regions and 5058 hypomethylated tile regions, representing 1080 and 3014 unique genes, respectively, in SNI animals whose methylation was affected by SAM treatment (Fig. 2B). Of these, 48 hypermethylated tiles and 142 hypomethylated tiles displayed robust differential methylation (adjusted *P* value < 1 × 10^−7^). The effect of SAM on control animals (Sham-Vehicle vs Sham-SAM animals) is reported in Supplemental Figure 1 (available at http://links.lww.com/PR9/A124).

A total of 1526 tile regions were differentially methylated in both the injury effect and SAM treatment comparisons. Of these 1526 tiles, 1415 tiles (92.7% of overlapping tiles), representing 1013 genes, had the SNI-driven changes in methylation reversed by SAM treatment. Sixty-four tiles (4.2%), representing 47 genes, experienced no reversal after SAM treatment, and 47 tiles (3.1%) display unclear reversal profiles and were excluded from further evaluation (Fig. 2C). Of the 1415 reversing tiles, 1048 tiles (755 genes) are initially hypermethylated during injury and experience hypomethylation after SAM treatment, whereas 367 tiles (258 genes) are hypomethylated during injury and hypermethylated after SAM treatment (Fig. 2D). The full list of differentially methylated genes identified in injury and SAM conditions, and those that undergo a reversal, can be found in Supplemental Tables 3, 4, and 5, respectively (http://links.lww.com/PR9/A117, http://links.lww.com/PR9/A118, and http://links.lww.com/PR9/A119).

We selected 4 genes (*Gfap, Adgrf2, Smc1b, and Kcng2*) for validation of differential methylation through pyrosequencing (Supplemental Figs. 2-5, available at http://links.lww.com/PR9/A124). Within the same tiled regions evaluated in the capture sequencing data, we found similar trends in DNA methylation in *Gfap, Adgrf2*, and *Smc1b*.

### 3.2. Specific gene ontology domains are affected during spared nerve injury, chronic S-adenosyl methionine administration, and reversal

We used gene ontology analysis to identify ontologies enriched for differentially methylated genes in the injury effect and SAM treatment conditions. Two hundred fourteen ontologies were enriched for the 3679 genes that were differentially methylated as a result of injury. Three hundred sixty-three ontologies were enriched for the 3913 differentially methylated genes resulting from SAM treatment of injury. We also identified 49 ontologies enriched for genes reversing their SNI-induced differential methylation through SAM treatment.

**Figure 3.**
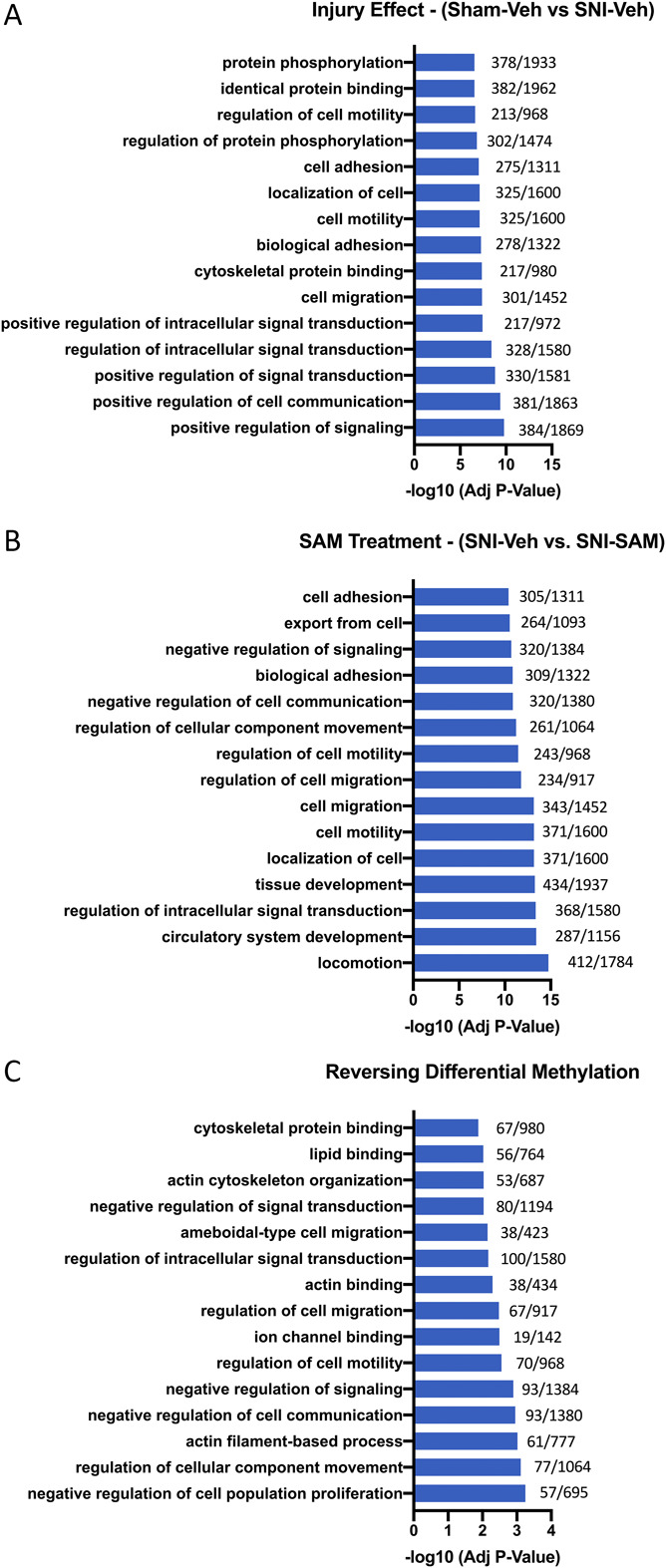
Functional domains and gene ontologies enriched in differentially methylated genes in SNI, SAM, and reversal conditions. The top 15 gene ontologies enriched for differentially methylated genes after (A) injury (3679 differentially methylated genes identify 214 enriched ontologies), (B) SAM treatment (3913 differentially methylated genes identify 363 enriched ontologies), and (C) reversal (1013 differentially methylated genes identifying 49 enriched ontologies). Displayed ontologies are filtered for a maximum size of 2000 genes and are from the Biological Processes or Molecular Function domains. Numbers on the right are differentially methylated genes/total genes in each ontology. Sham-Vehicle: n = 3, SNI-Vehicle: n = 4, and SNI-SAM: n = 4. SNI, spared nerve injury; SAM, S-adenosyl methionine.

To highlight the top 15 gene ontologies, we limited ontology size to 2000 genes and sorted by adjusted *P* value. The top 15 enriched ontologies in injury are predominantly involved in signaling and intracellular signal transduction, cell migration, cytoskeletal protein binding, and biological adhesion (Fig. 3A). The top 15 enriched ontologies after SAM treatment are involved in locomotion and cell motility, circulatory system and tissue development, intracellular signal transduction, and cellular transport (Fig. 3B). The top 15 enriched ontologies that were affected by injury and reversed by SAM treatment are involved in locomotion and cell motility, actin cytoskeleton, intracellular signal transduction, and cellular transport (Fig. 3C). The full list of gene ontologies enriched for differentially methylated genes in injury and SAM conditions and gene ontologies enriched for genes that undergo a reversal of differential methylation can be found in Supplemental Table 6, 7, and 8, respectively (http://links.lww.com/PR9/A120, http://links.lww.com/PR9/A121, and http://links.lww.com/PR9/A122).

### 3.3. Pain-related genes undergo changes and reversals in DNA methylation as a result of spared nerve injury and S-adenosyl methionine treatment

**Figure 4.**
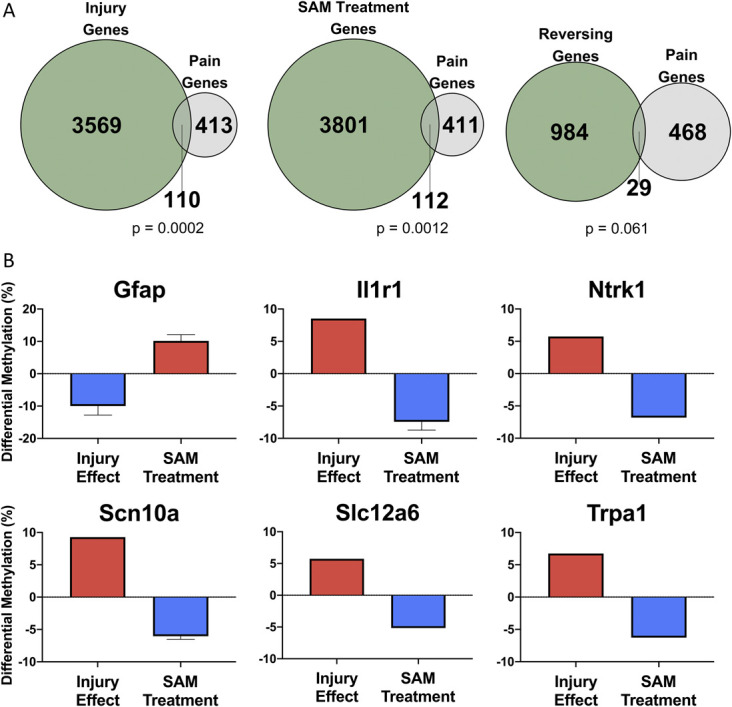
Pain-related differentially methylated genes are detected in injury, SAM, and reversal conditions. (A) Injury and SAM treatment conditions were significantly enriched for pain-related differentially methylated genes, and a trend (*P* = 0.061) was observed for the reversal condition. Displayed are the number of differentially methylated genes (green), the number of pain genes detected in this condition (gray), and the number of differentially methylated pain genes (overlap) detected. Hypergeometric test. (B) Differential methylation of 6 pain-related genes is shown, where displayed differential methylation is the average of all identified tiles per gene. Error bars represent the standard error of differential methylation when multiple tiling regions are annotated to the gene of interest. *Gfap*: glial fibrillary acidic protein; *Il1r1*: interleukin receptor 1, type 1; *Ntrk1*: neurotrophic receptor tyrosine kinase 1; *Scn10a*: sodium voltage-gated channel alpha subunit 10; *Slc12a6*: K-Cl cotransporter 3; *Trpa1*: transient receptor potential cation channel subfamily A, member 1. Pain genes have been previously identified by Ultsch et al. 2013.^66^ Sham-Vehicle: n = 3, SNI-Vehicle: n = 4, and SNI-SAM: n = 4. SNI, spared nerve injury; SAM, S-adenosyl methionine.

To identify pain-related genes, differentially methylated genes were compared with a curated list of pain-related genes identified by Ultsch et al.^66^ Spared nerve injury resulted in differential methylation that was enriched for pain-related genes, with 110 differentially methylated pain-related genes identified by Ultsch (*P* = 0.0002; hypergeometric test). In the SAM treatment effect, 112 differentially methylated pain genes were identified from the Ultsch list (*P* = 0.0012; hypergeometric test). We identified 29 pain-related genes that undergo a reversal in their methylation status as a result of SAM treatment of SNI (*P* = 0.061; hypergeometric test) (Fig. 4A). These genes include the TrkA receptor *Ntrk1*, Na_v_1.8 channel *Scn10a*, and glutamate transporter *Slc12a4* (Fig. 4B). The full list of pain-related differentially methylated genes can be found in Supplemental Table 9 (available at http://links.lww.com/PR9/A123).

## 4. Discussion

We previously demonstrated that SAM chronic treatment attenuates nerve injury–related mechanical sensitivity as well as injury-related cognitive deficit.^27^ To identify novel avenues of treatment against chronic pain, we examined the mechanisms underlying SAM-related attenuation of chronic pain. Our current study demonstrates that SAM treatment reverses chronic pain–induced changes in DNA methylation in the mouse frontal cortex and identifies associated genes and gene pathways.

First, we confirmed previous findings of differential methylation many months after nerve injury^39,63,64^ and in response to chronic systemic SAM administration.^27^ More than 3500 genes were differentially methylated after either injury or SAM treatment, indicating that both states are capable of inducing widespread cortical methylation changes. Second, we showed the reversal of injury-related differential methylation after SAM treatment is concurrent with pain attenuation. This is consistent with work from other domains where reduction of a disease state with treatment is associated with reversing methylation patterns.^38,68^ Third, we identified pain-related genes and gene ontologies that are enriched for genes that reverse differential methylation as a result of intervention. These data support a role for the epigenome in the maintenance of chronic pain and advance epigenetic modulators such as SAM as a novel approach to treat chronic pain.

### 4.1. Methylation in response to injury and SAM in the frontal cortex

Both neuropathic pain and SAM treatment are capable of inducing widespread and highly significant changes in frontal cortex methylation, with each condition inducing differential methylation in nearly 17% of the ∼22,500 genes examined. Regions were observed with robust differential methylation of up to 40% and adjusted *P* values of less than 1 × 10^−7^. These widespread changes demonstrate not only the global impact of chronic pain but also the responsivity of the methylome to environmental influences, including here, a nutritional supplement.

At 7 months postinjury, there was a trend towards hypermethylation when observing the effect of SNI. This is consistent with previous reports of hypermethylation in the frontal cortex at multiple time points after SNI^64^ or 3 weeks after spinal nerve ligation.^26^ Injury-induced hypermethylation would imply decreased gene repression in response to injury. In studies of mRNA expression after injury, Alvarado et al. 2013 found decreases in mRNA expression in mouse PFC 6 months after SNI (278 upregulated genes to 367 downregulated genes),^2^ and Descalzi et al. 2017 found similar results 2.5 months after SNI in mouse medial prefrontal cortex (179 upregulated to 204 downregulated genes).^20^ Although this may indicate a general trend, both hypermethylated and hypomethylated genes must be considered when identifying dysregulated functions.

In the current study, a trend towards promoter hypomethylation after SAM treatment of SNI animals was observed. This was unexpected because SAM is the primary methyl donating substrate for DNMTs and would therefore be expected to increase methylation. We previously established that chronic administration of SAM significantly increases global methylation in PFC postinjury.^27^ There are a number of explanations why systemic SAM treatment might decrease methylation in promoters while increasing global methylation. SAM is not only a methyl donor to DNMTs but also the primary methyl donor to most methyltransferase enzymes including histone methyltransferases and catechol-O-methyltransferase.^23,58,62,65^ Histone methylation at different histone tail positions can recruit chromatin-modifying complexes, resulting in either open or closed chromatin and corresponding changes in DNA methylation.^15^ Therefore, increases in SAM availability could alter histone methylation patterns with increased H3K4 or H3K36 methylation associated with open chromatin and hypomethylation and increased H3K9 or H3K27 methylation leading to closed chromatin and hypermethylation.^15,40^ Therefore, increased SAM could disrupt the methylation equilibrium at a genome-wide level, inducing direct and indirect effects. Previous genome-wide analyses of DNA methylation after SAM treatment revealed both hypomethylation and hypermethylation.^72^ Moreover, the SAM metabolite S-adenosyl homocysteine is an inhibitor of most methyltransferases^17,19^; therefore, increased administration of SAM may lead to an accumulation of S-adenosyl homocysteine.

### 4.2. Reversal of methylation after S-adenosyl methionine treatment in the frontal cortex in injured animals

Changes in DNA methylation in response to external stimuli are well established, and a reversal of disease-related or condition-related DNA methylation after successful treatment has been reported.^38,68,73^ This is the first study to investigate epigenome-wide reversals of DNA methylation associated with a chronic pain condition after effective attenuation of pain. We demonstrate that an intervention that attenuates neuropathic pain–related mechanical hypersensitivity also reverses injury-driven changes in DNA methylation at hundreds of gene promoters.

In this study, nearly all tiles that were differentially methylated in both injury and SAM (1415/1526) were reversed by the treatment. This is notable as it represents the reversion of more than 1000 genes towards the uninjured methylation state after a successful intervention. Proportionally, this represents ∼5% of the mouse genome and ∼25% of genes that were differentially methylated postinjury. This subset of genes may play critical roles in mediating chronic pain's impact on the brain. These genes are enriched in domains of intracellular signalling, actin and cytoskeletal structure, cell migration, and ion channel binding; all of these have been hypothesized to contribute to chronic pain–related decreases in gray matter and subsequent recovery after treatment in humans.^30,36,47^ However, although SAM treatment is capable of attenuating pain-related differential methylation, in general it was not sufficient to return altered DNA methylation to the equivalent of a nonchronic pain state.

### 4.3. Differentially methylated functional domains and pain-related genes in injury and S-adenosyl methionine

Consistent with previous studies, differentially methylated genes are enriched for domains of intracellular signalling, cell motility and locomotion, cellular adhesion, and cytoskeletal formation in the frontal cortex after nerve injury.^39,64^ After SAM treatment of injured animals, differentially methylated genes are enriched in similar functional domains as observed after injury, implying that SAM acts on pathways that are reprogrammed by injury and the resulting chronic neuropathic pain. This convergence advocates for further exploration of epigenetic drugs such as SAM as potential therapeutics. This convergence also implicates cortical DNA methylation in the maintenance of long-term chronic pain.

### 4.4. Limitations

This study has limitations. First, the low sample size of 3 to 4 animals and permissive adjusted *P* value of 0.1 dictate that these results should be considered exploratory. Second, although the focus is on promoter region methylation, methylation at distal enhancers and within the gene body are also known to play a role in regulating gene transcription.^4,5,74^ Although promoter region methylation is a dominant mechanism of DNA methylation regulation, other mechanisms influence gene transcription, translation, and ultimately expression. Third, the chronic systemic administration of SAM may affect chronic neuropathic pain through mechanisms unrelated to DNA methylation. As stated above, SAM also is a methyl donor for histone methyltransferases and COMT, both of which have well-established impacts on pain sensitivity. Fourth, our study contained males alone; therefore, our conclusions do not account for sex differences in response to chronic pain or in response to exposure to SAM. Fifth, the impact of SAM on the transcriptome and proteome was not examined. Future studies incorporating measurement of mRNA and protein levels are needed to determine the downstream impact of differential DNA methylation. Finally, the phenomena described here are correlational; further research is needed to explore causal relationships between frontal cortex methylation and chronic pain behaviour. While we recognize these limitations, we believe these results provide an important first look into the frontal cortical plasticity in DNA methylation associated with the successful treatment for chronic pain.

## 5. Conclusions

Our findings provide first evidence for epigenome-wide reversal of pain-induced differential DNA methylation after treatment in an animal model of chronic pain by an epigenetic modulator. These changes occurred in domains of intracellular signaling, cell motility and locomotion, and cytoskeletal structure, reflecting potential changes in neuroinflammation and synaptic pruning and formation. These data suggest that DNA methylation may contribute to chronic pain persistence and to the cortical recovery observed after therapeutic interventions. These findings provide crucial insight into the relationship between chronic pain and the frontal cortex and call for increased exploration of epigenetic drugs, such as SAM, for the treatment of chronic pain.

## Disclosures

The authors have no conflicts of interest to declare.

## Appendix A. Supplemental digital content

Supplemental digital content associated with this article can be found online at http://links.lww.com/PR9/A115, http://links.lww.com/PR9/A116, http://links.lww.com/PR9/A124, http://links.lww.com/PR9/A117, http://links.lww.com/PR9/A118, http://links.lww.com/PR9/A119, http://links.lww.com/PR9/A120, http://links.lww.com/PR9/A121, http://links.lww.com/PR9/A122 and http://links.lww.com/PR9/A123.

## Supplementary Material

SUPPLEMENTARY MATERIAL
